# Active Targeting of P-Selectin by Fucoidan Modulates the Molecular Profiling of Metastasis in Docetaxel-Resistant Prostate Cancer

**DOI:** 10.3390/md20090542

**Published:** 2022-08-23

**Authors:** Chang-Hsun Ho, Mei-Lin Chen, Hau-Lun Huang, Chih-Jen Lai, Chih-Hsin Liu, Chih-Pin Chuu, Yu-Hsin Lin

**Affiliations:** 1 Department of Anesthesiology, Show Chwan Memorial Hospital, Changhua 50008, Taiwan; 2 Department of Pharmacy, Cheng Hsin General Hospital, Taipei 11220, Taiwan; 3 Department of Pharmacy, National Yang Ming Chiao Tung University, Taipei 11221, Taiwan; 4 Institute of Cellular and System Medicine, National Health Research Institutes, Miaoli 35053, Taiwan; 5 Medical Device Innovation and Translation Center, National Yang Ming Chiao Tung University, Taipei 11221, Taiwan; 6 Department of Medical Research, China Medical University Hospital, China Medical University, Taichung 40402, Taiwan

**Keywords:** prostate cancer, docetaxel resistant, fucoidan, metastasis-inhibiting signaling pathway, synergistic effects

## Abstract

The standard of care for prostate cancer (PCa) is androgen deprivation therapy (ADT). Although hormone-sensitive PCa is curable by ADT, most conditions progress to castration-resistant prostate cancer (CRPCa) and metastatic CRPCa (mCRPCa). Front-line docetaxel has been administered to patients with CRPCa and mCRPCa. Nevertheless, docetaxel resistance after half a year of therapy has emerged as an urgent clinical concern in patients with CRPCa and mCRPCa. We verified the mechanism by which docetaxel-resistant PCa cells (DU/DX50) exhibited significant cell migration and expression of malignant tumor-related proteins. Our study shows that the biological activity of fucoidan has an important application for docetaxel-resistant PCa cells, inhibiting IL-1R by binding to P-selectin and reducing the expression levels of NF-κB p50 and Cox2 in this metastasis-inhibiting signaling pathway. Furthermore, the combined treatment of fucoidan and docetaxel showed significant anticancer and synergistic effects on the viability of DU/DX50 cells, which is relevant for overcoming the current limitations and improving treatment outcomes. Overall, fucoidan-based combination chemotherapy may exert beneficial effects and facilitate the treatment of docetaxel-resistant PCa.

## 1. Introduction

Prostate cancer (PCa) is the second most frequently diagnosed cancer worldwide, causing nearly 10 million deaths in 2020 or nearly 1 in 6 deaths [1,2,3]. According to Jemal et al., North America, Oceania, and Western Europe have the highest incidence rate of PCa [4]. The data reveal a marked rise in prostate-specific antigen detection and disease incidence in developing countries, making PCa the leading cancer in men [5,6]. The standard treatment for advanced PCa is androgen deprivation therapy (ADT) [7,8]. Despite PCa progression to the metastatic stage, blocking androgen receptor activation remains the mainstay of therapy [7,8,9]. Although hormone-sensitive PCa is curable with ADT, most patients progress to castration-resistant prostate cancer (CRPCa) and metastatic CRPCa (mCRPCa) [7,8,9]. Front-line docetaxel treatment is administered to patients with CRPCa and mCRPCa to improve survival [9,10]. Docetaxel is a chemotherapeutic agent belonging to the taxane class of drugs [11,12]. Docetaxel-based chemotherapy has shown survival benefits and has emerged as the primary treatment for CRPCa [13]. Nevertheless, docetaxel resistance after half a year of therapy has emerged as an urgent clinical concern in patients with CRPCa and mCRPCa [14,15].

ATP-binding cassette (ABC) transporters are a family of transporters responsible for the expulsion of multiple drugs from the cells at the expense of ATP hydrolysis, leading to drug resistance and low drug bioavailability [16]. The major multidrug-resistance-related proteins 1-5 (MRP_1-5_/ABCC_1-5_, from the ABCC subfamily) and ABC transporters involved in the efflux of toxic substrates are P-glycoproteins (Pgp/MDR_1_/ABCB_1_, from the ABCB subfamily) or breast cancer resistance proteins (BCRP/ABCG_2_, from the ABCG subfamily) [17]. ABCB1 is mainly involved in the efflux of unmodified exogenous compounds, whereas the substrates of ABCC1-5 and ABCG2 transporters are mainly glutathione-, glucuronide-, or sulfate-conjugated metabolites [18]. The upregulation of ABCB1 leads to the relapse of docetaxel resistance and poor survival in ovarian cancer patients [19]. Previous studies have shown that the overexpression of ABCB1 confers chemoresistance and is directly associated with a higher tumor stage and grade in PCa [20]. In chemotherapy, following the administration of a drug, tumors in a high number of patients develop drug resistance. At present, chemotherapy failure commonly occurs due to resistance-related cancer invasion and metastasis [21,22]. Therefore, it is of great importance to study the mechanisms and pathways of drug resistance and PCa metastasis and identify useful therapeutic targets to advance current treatment modalities [23].

Fucoidan, sourced from various matrices of brown seaweed, is primarily composed of a complex sulfated polysaccharide [24,25]. It mainly consists of sulfated L-fucose with varying proportions of mannose, galactose, glucose, xylose, and uronic acid, depending on the species of brown algae [26]. Fucoidan has been reported to exhibit anticancer, antimetastatic, antioxidant, anti-inflammatory, anticoagulant, antithrombotic, antilipidemic, antidiabetic, and antiviral activities [27]. In parallel, the utilization of natural resources with chemotherapeutic cytotoxicity for cancer treatment is a recently developed technology in cancer research. Fucoidan is a natural compound that can retard tumor growth, eradicate cancer cells, and synergize with anticancer chemotherapeutic agents [28]. As a polysaccharide ligand of adhesion P-selectin molecule, it exhibits antiproliferative properties [29]. Furthermore, the involvement of P-selectin in the adhesion of cancer cells to the endothelium suggests that the activated endothelium in vivo has a pro-metastatic function [30,31]. In the current study, we aimed to analyze the biological activities and functions of fucoidan, which has important implications for its application on the metastasis of docetaxel-resistant PCa cells, thereby endeavoring to maximize their combined therapeutic effect and provide synergistic anticancer effects.

## 2. Results

### 2.1. Docetaxel-Resistant PCa Cells Exhibit High Motility and *Malignancy-Associated Protein* Expression

**Figure 1:**
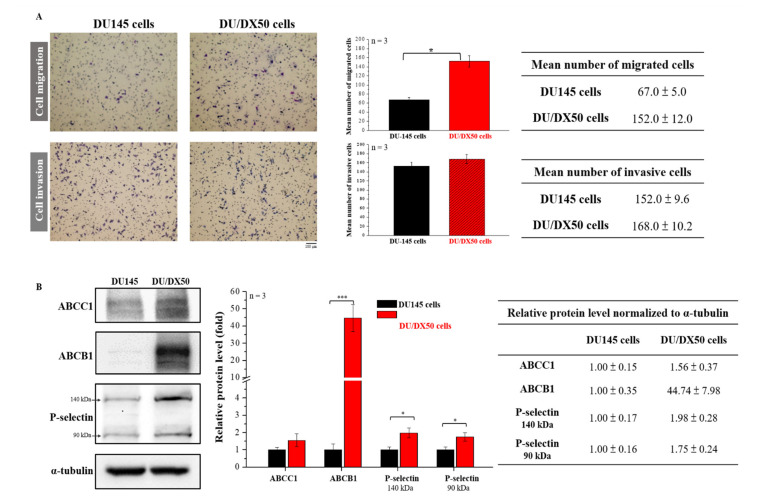
Determination of the migration and invasion ability and examination of the expression of drug efflux proteins with docetaxel-resistant PCa cells. (**A**) The motility of DU145 and DU/DX50 cells was examined using a Transwell assay. Cells were counted and quantified. (**B**) Protein expression levels of ABCC1, ABCB1, and P-selectin were determined using Western blotting. α-Tubulin was used as a loading control. Quantification of protein abundance in DU145 and DU/DX50 cells. Asterisks * and *** represent statistically significant differences, with *p*-values < 0.05 and < 0.001.

To evaluate the migration and invasion abilities of parental as well as docetaxel-resistant PCa cells, we examined the percentage of migration and invasion in PCa cell line DU145 (ATCC^®^ HTB-81™) and 50 nM docetaxel resistance of DU/DX50 cells using the Transwell assay. Representative profiles and quantitative analyses are presented in Figure 1A. The quantified results show that the number of migrated DU/DX50 cells is significantly higher than DU145 cells (152 ± 12 cells vs. 67 ± 5 cells). However, there are no differences in the number of invasive cells. To confirm the drug-resistance status of DU/DX50 cells, we examined the expression levels of drug efflux protein families (ABCC1 and ABCB1) and P-selectin using Western blotting. Notably, the data revealed that the level of ABCB1 in DU/DX50 cells was over 40-fold higher than that in DU145 cells. Additionally, P-selectin was overexpressed and ABCC1 expression was slightly increased in DU/DX50 cells (Figure 1B). Taken together, these data demonstrate that DU/DX50 cells have high migration and drug-resistance abilities.

### 2.2. Effects of Fucoidan on P-Selectin Binding and Cellular Uptake

**Figure 2:**
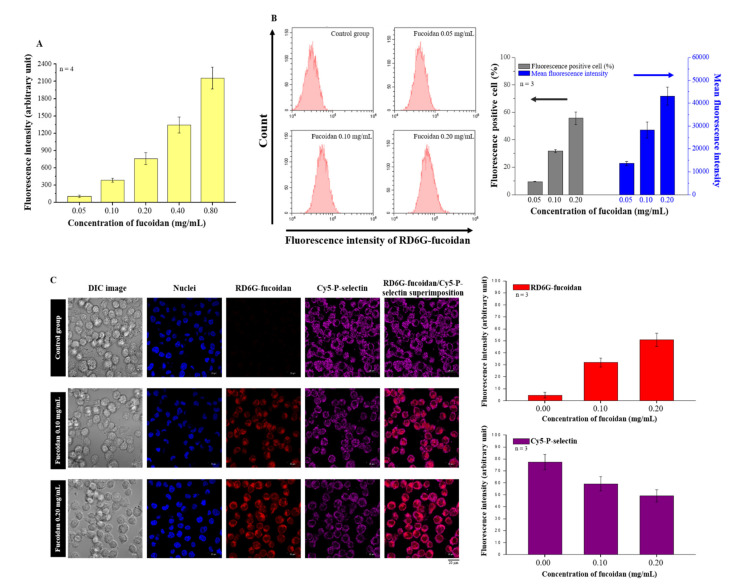
Evaluating the P-selectin binding ability of fucoidan. (**A**) Assessment of the P-selectin binding ability of fucoidan using in vitro binding assay. (**B**) Flow cytometry results reveal the cellular uptake of fucoidan in a concentration-dependent manner. (**C**) Images of Cy5-P-selectin and RD6G-fucoidan from CLSM and quantified data.

Fucoidan is a ligand of P-selectin. In this study, we examined the binding ability of fucoidan and P-selectin using an in vitro binding assay, and we observed that the binding ability of fucoidan and P-selectin increased in a concentration-dependent manner (Figure 2A). Subsequently, we confirmed the cellular uptake of fucoidan using flow cytometry. When the concentration of rhodamine 6G (RD6G)-fucoidan was increased, we observed more fluorescent-positive cells and mean fluorescence intensity (Figure 2B). Moreover, confocal laser scanning microscopy (CLSM) images also illustrated the co-localization of RD6G-fucoidan and Cy5-P-selectin in DU/DX50 cells (Figure 2C). These results suggest that fucoidan accumulation is closely correlated with P-selectin expression. Interestingly, we observed that the levels of P-selectin decreased following fucoidan treatment in DU/DX50 cells (Figure 2C).

### 2.3. Fucoidan Reduces the Expression of the ABC Transporter Family and P-Selectin in Docetaxel-Resistant DU/DX50 Cells

**Figure 3:**
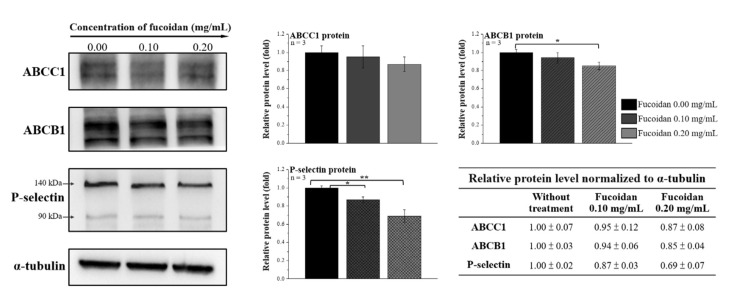
Fucoidan altered the expression of ABC transporter and P-selectin in docetaxel-resistant PCa cells. The protein expression levels of ABCC1, ABCB1, and P-selectin were determined by Western blotting in DU/DX50 cells treated with fucoidan. α-Tubulin was used as a loading control. Asterisks * and ** represent statistically significant differences, with *p*-values < 0.05 and < 0.01.

ABCB1 overexpression is directly correlated with chemoresistance and tumor progression in PCa. Moreover, P-selectin is involved in tumor metastasis. We speculated that fucoidan treatment alters the levels of ABC transporters and P-selectin in DU/DX50 cells. Therefore, the protein expression levels of ABCC1, ABCB1, and P-selectin in DU/DX50 cells treated with fucoidan were determined by Western blotting. Fucoidan treatment significantly reduced ABCB1 expression at a concentration of 0.2 mg/mL. In addition, P-selectin expression was notably downregulated in both the 0.1 and 0.2 mg/mL groups (Figure 3), correlating with fucoidan to P-selectin images (Figure 2C).

### 2.4. Fucoidan Attenuates the Abilities of Migration and Invasion in Docetaxel-Resistant DU/DX50 Cells

**Figure 4:**
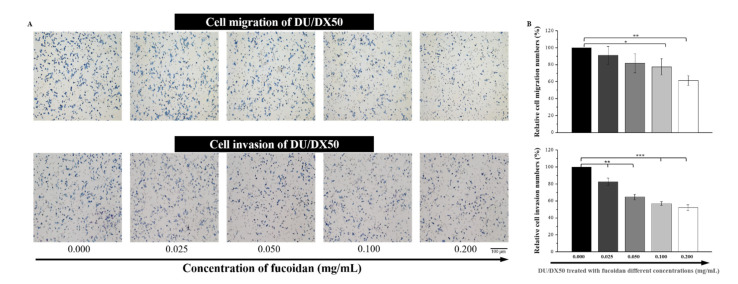
Fucoidan retards cell mobility in docetaxel-resistant PCa cells. (**A**) Images of DU/DX50 cells treated with fucoidan in Transwell migration and invasion assays. (**B**) Quantification of mobile cells in the Transwell migration and invasion assays. Asterisks *, ** and *** represent statistically significant differences, with *p*-values < 0.05, < 0.01 and < 0.001, respectively.

Drug-resistant DU/DX50 cells presented higher motility than parental DU145 cells. Fucoidan has been reported to have an anti-metastatic function. Therefore, we evaluated the migration and invasion abilities of docetaxel-resistant DU/DX50 cells under fucoidan treatment. With increasing concentrations of fucoidan, we observed that the number of migrated cells significantly decreased under 0.1 mg/mL to 0.2 mg/mL of fucoidan treatment. Notably, the relative number of invasion cells in the Transwell invasion assay were 100.00 ± 0.00%, 82.65 ± 4.45%, 64.74 ± 3.08%, 56.95 ± 2.19% and 52.29 ± 3.14%, corresponding to fucoidan treatment concentrations of 0.000, 0.025, 0.052, 0.100 and 0.200 mg/mL, respectively. Thus, fucoidan treatment significantly reduced the migration and invasion of docetaxel-resistant PCa cells in a concentration-dependent manner (Figure 4).

### 2.5. Expression of Hypothesized Signal Transduction Pathways and Metastasis-Related Protein in Fucoidan Treated Docetaxel-Resistant DU/DX50 Cells

**Figure 5:**
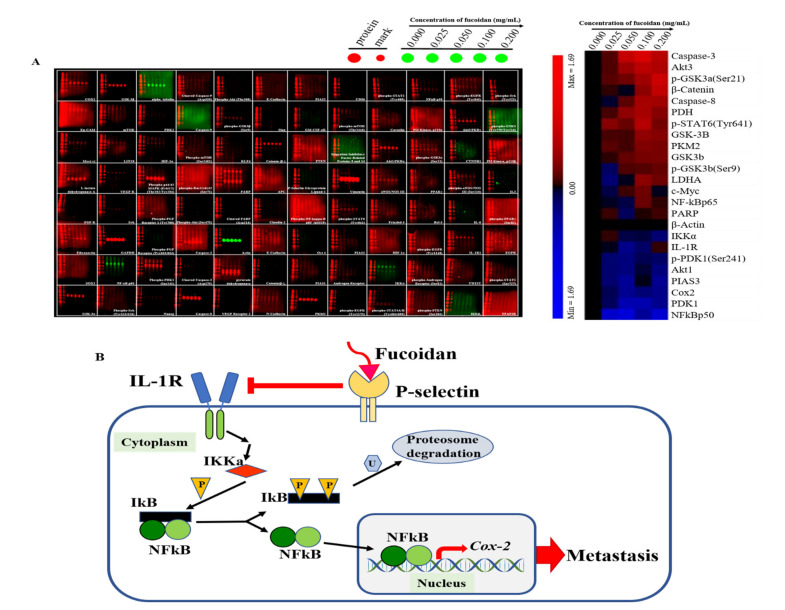
Metastasis-related protein profiles in PCa cells treated with fucoidan and the related proposed signaling pathway. (**A**) Modulation of protein abundance in DU/DX50 cells was determined using MWA and different antibodies. Representative MWA images and their results via heatmap are shown. (**B**) Schematic diagram showing the inhibition of the expression of IL-1R in the membrane and IKKα in the cytoplasm, leading to the expression of the precursor form of NF-kB (p105) and a decrease in the active form of NF-kB (p50), resulting in a decrease in Cox2 expression and a reduction in metastasis of PCa cells.

**Figure 6:**
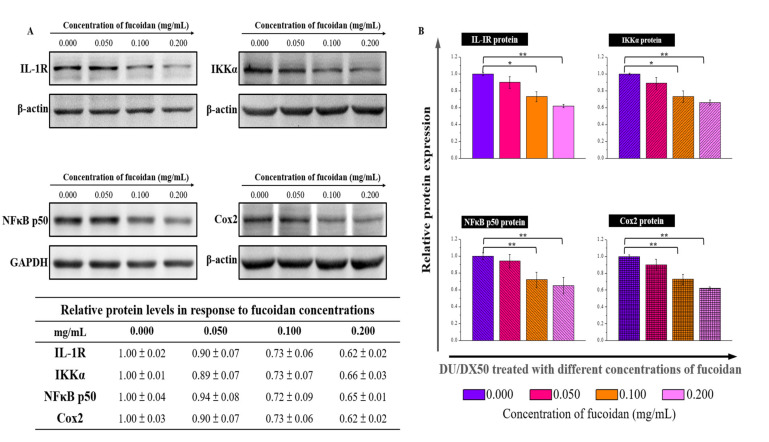
Metastasis-related protein expression in fucoidan-treated PCa cells determined by traditional Western blotting. (**A**) Protein levels of IL-1R, IKKα, NFkB p50, and Cox2 in DU/DX50 cells following fucoidan treatment were shown by Western blotting. GAPDH and α-actin were used as loading controls. (**B**) Quantification of DU/DX50 protein expression after treatment with fucoidan. Asterisks * and ** represent statistically significant differences, with *p*-values < 0.05 and < 0.01.

Since fucoidan-treated DU/DX50 cells might express unique protein profiles associated with metastasis, we performed MWA to verify our hypothesis. The results of the heatmap indicate that IL-1R, IKKα, NFκB p50, and Cox2 expression levels are downregulated compared to the control group without sample treatment (Figure 5A). In the current study, we proposed a possible signaling pathway by which fucoidan inhibited the motility of drug-resistant DU/DX50 cells (Figure 5B). In fucoidan-treated DU/DX50 cells, fucoidan interacted with P-selectin, which induced the downregulation of IL-1R and caused a reduction in IKKα expression. Meanwhile, IKKα reduction decreased the phosphorylation of IκBα, inactivated NFκB, and resulted in decreased Cox2 expression by blocking this signaling pathway to inhibit metastasis. To verify the expression levels of the metastatic proteins obtained from the microwestern array (MWA), we performed Western blotting. The results indicate that the expression levels of IL-1R, IKKα, NF-κB p50, and Cox2 diminish in response to increasing concentrations of fucoidan (Figure 6).

### 2.6. Anti-Cancer Effects of Fucoidan and Docetaxel and the Synergistic Effect of the Combination of Fucoidan/Docetaxel on Docetaxel-Resistant DU/DX50 Cells

**Figure 7:**
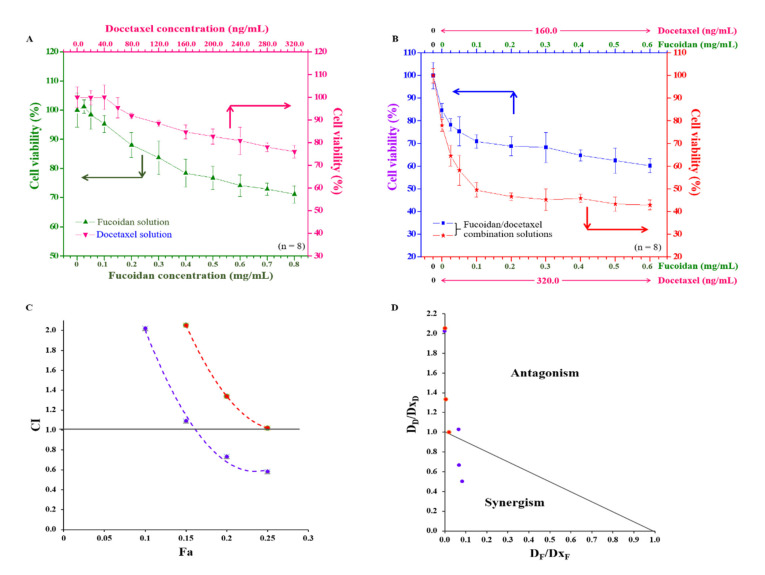
The effect of single-use or combination of fucoidan and docetaxel on the viability of PCa cells. (**A**) Viability of DU/DX50 cells in response to fucoidan (green line) or docetaxel (pink line) treatment. (**B**) Viability of DU/DX50 cells treated fucoidan/docetaxel combination solution with various fucoidan concentrations ranging from 0.0–0.6 mg/L mixed with docetaxel concentrations of 160.0 (blue line) or 320.0 ng/mL (red line) solutions. (**C**) Combination index fraction affected (CI–Fa) plot at fixed docetaxel concentration of 160 ng/mL (purple line) or 320.0 ng/mL (orange line) in combination with fucoidan; CI < 1.0 indicates synergy; CI = 1.0 indicates additive effect, and CI > 1.0 indicates antagonism of the combined drug effect. (**D**) Isobologram. D_F_ and D_D_ are the concentrations of fucoidan and docetaxel used in combination to achieve x% drug effect. Dx_F_ and Dx_D_ are the concentrations at which a single drug achieves x% drug effect. The dots below the slashes indicate the synergistic effects.

We estimated the potential inhibitory effects of fucoidan or docetaxel alone as well as analyzed the synergy of the combinatorial treatment of fucoidan/docetaxel on docetaxel-resistant PCa cell viability. These drugs alone inhibited cell proliferation in a concentration-dependent manner, but only at concentrations of 0.8 mg/mL and 320.0 ng/mL of fucoidan and docetaxel, respectively, showing a 30% maximal inhibitory concentration (IC_30_) of DU/DX50 cells (Figure 7A). Additionally, the anti-cancer effects of fucoidan/docetaxel as a combination treatment were compared, where docetaxel at a concentration of 160 ng/mL or 320 ng/mL was combined with fucoidan at various concentrations (ranging from 0.0 to 0.6 mg/mL). The cell viability decreased from 84.67 ± 3.02% to 60.21 ± 3.09% or 78.06 ± 2.76% to 42.91 ± 2.19% for each concentration of docetaxel, respectively (Figure 7B). In addition, fucoidan/docetaxel treatment had a potent synergistic effect on cell viability, and at a fixed docetaxel concentration of 160 ng/mL in combination with fucoidan, the changes in the combination index (CI) values of IC_10_, IC_15_, IC_20_, and IC_25_ were 1.01, 0.51, 0.33 and 0.25, respectively (Figure 7C). Plots and isobolograms of CI values versus fraction affected (Fa) are presented in Figure 7C,D.

## 3. Discussion

The efflux drug transporter was overexpressed in docetaxel-resistant cells [15]. The P-glycoprotein-encoding gene, *ABCB1*, an efflux drug transporter, plays a canonical role in docetaxel resistance in CRPCa [15]. Docetaxel-resistant PCa cells highly express the AKT-dependent drug transporter ABCB1 and demonstrate cell migration and invasion abilities [14]. This biomarker enhanced cell migration and invasion. Therefore, we analyzed the following events: first, the ability of invasion and migration by the Transwell assay, and second, the expression of drug efflux transporters and P-selectin by Western blotting. Our results reveal that drug-resistant DU/DX50 cells have a two-fold-higher number of migrated cell numbers compared to parental human PCa cells (DU145) (Figure 1A). The quantification of protein expression also confirmed that drug resistance was present in DU/DX50 cells (Figure 1B). Elevated levels of P-selectin have been observed in many types of human cancer cells. In the present study, we observed that the DU/DX50 cell line expressed more P-selectin than parental DU145 cells (Figure 1B).

Furthermore, fucoidan interacts with the P-selectin of its carbohydrate ligands [32]. In this study, we confirmed the binding between fucoidan and P-selectin using a binding assay (Figure 2A). Fucoidan protects against acute pancreatitis in a mouse model because it is a P- and L-selectin inhibitor that also inhibits other adhesion molecules on the surface of tumor cells, such as integrins [28,33]. As shown in Figure 2C, the colocalized images and quantitative analysis of P-selectin levels diminished in response to the increased concentration of fucoidan. Moreover, we observed a similar phenomenon by Western blotting in DU/DX50 cells, where the expression level of P-selectin significantly decreased in response to fucoidan (Figure 3), suggesting that fucoidan exerted an inhibitory effect on P-selectin. In this study, we observed that fucoidan also reduced the migration and invasion of DU/DX50 cells (Figure 4). P-selectin can induce metastasis by increasing the number of circulating tumor cells that adhere to the endothelium in distant organs [34]. Therefore, we speculated that fucoidan inhibits the expression of P-selectin, thereby attenuating cell migration and invasion.

According to the previous studies, fucoidan has an antimetastatic effect on a human lung cancer cell line [35]. Fucoidan treatment also resulted in the apoptosis of breast cancer cells due to caspase-8 activation and induced caspase 3 and PARP in human lung cancer cells [36,37]. In this study, we observed that fucoidan could inhibit cell migration and invasion, and thus, we focused on clarifying the signaling pathway related to metastasis. To clarify the mechanism by which fucoidan influences cell migration and invasion, we examined the regulatory levels of metastasis-related proteins by MWA (Figure 5A). IL-1 regulatory pathways increase drug resistance and tumor survival by inducing the release of proinflammatory cytokines [38]. IKKα and NF-κB p50 are involved in cancer cell proliferation and metastasis [39]. The activation of Cox2 promotes tumor growth and resistance to chemotherapy and radiotherapy [40]. Our results reveal that the protein levels of IL-1R, IKKα, NF-κB p50, and Cox2 are downregulated with an increased concentration of fucoidan. According to the results presented in the heatmap, cancer cell migration and invasion attenuated through the binding effect between fucoidan and P-selectin, resulting in the downregulation of the IL-1R signaling pathway, including reduced levels of NFκB p50 and Cox-2 (Figure 5B). To further confirm the MWA results, the levels of these proteins in fucoidan-treated DU/DX50 cells were analyzed by Western blotting. We demonstrated that the protein levels of IL-1R, IKKα, NFκB p50, and Cox2 decreased in response to an increased concentration of fucoidan (Figure 6). Moreover, we also demonstrated that the combined therapeutic effect of docetaxel and fucoidan affected cell viability and presented a potent synergistic antiproliferative effect (Figure 7). We believe that fucoidan is a potent chemotherapeutic agent for the treatment of docetaxel-resistant PCa cells. In the future, prospective studies may demonstrate the beneficial effects of standalone fucoidan and fucoidan-based combined interventions in animal models compared to other chemotherapeutic agents.

## 4. Materials and Methods

### 4.1. Transwell Migration, Invasion, and Western Blotting Arrays on Docetaxel-Resistant PCa Cells

The PCa cell line DU145 was purchased from American Tissue Culture Collection (ATCC) (Manassas, VA, USA). Docetaxel-resistant sublines DU/DX50 were developed by recurrently exposing DU145 cells to gradually increasing concentrations of docetaxel in Prof. Chih-Pin Chuu’s laboratory (Institute of Cellular and System Medicine, National Health Research Institutes, Miaoli, Taiwan). Both cell lines were grown in Roswell Park Memorial Institute (*RPMI*)-1640 medium (Thermo Fisher Scientific, Logan, UT, USA) supplemented with 10% fetal bovine serum (FBS), penicillin (100 units/mL), and streptomycin (0.1 mg/mL) (Lonza, Walkersville, MD, USA). DU/DX50 cells were maintained with 50 nM docetaxel, and all the cells were propagated in standard cell culture conditions of 37 °C, 95% humidity_,_ and 5% CO_2_ [41]. After reaching confluence, the cells were detached from the dishes using 0.25% trypsin/ethylenediaminetetraacetic acid (EDTA), and trypsin was removed by centrifugation. The cell pellet was resuspended in a fresh medium for further experiments.

The migration ability of DU145 and DU/DX50 cells was examined using 8 mm pore-size 24-well Transwell dishes (BD Biosciences, San Jose, USA) or invasion assay using Growth-Factor-Reduced BD BioCoat Matrigel invasion chambers following the manufacturer’s instructions [42]. The cells were seeded in the upper chamber in a serum-free RPMI medium (500 µL) at a density of 1 × 10^4^, while complete medium (1000 µL) was added to the lower chamber of the plate. The cells were incubated for 16 h, and those on the lower surface of the polyester membrane were fixed with ice-cold methanol and stained with 0.2% crystal violet for 10 min [43,44]. Cotton-tipped swabs were used to remove cells from the upper side of the filters, after which the cells were washed with deionized water. Five fields of each well were randomly selected and imaged, and the cells were counted at the bottom of the filters using an optical microscope.

Differential DU145 and DU/DX50 cells were seeded at a density of 6.0 × 10^5^/10 cm dishes and cultured for 72 h. The cells were then lysed with radioimmunoprecipitation assay (RIPA) buffer containing phosphatase inhibitors, and proteins were quantified using the Bradford protein assay. Equal amounts of protein were separated by 10% sodium dodecyl sulfate (SDS) polyacrylamide gel electrophoresis. The proteins separated from the gel were transferred to polyvinylidene difluoride membranes, followed by blocking with defatted dry milk in phosphate-buffered saline (PBS) for 1 h. The proteins were detected using the following primary antibodies: anti-ABCC1, anti-ABCB1, anti-P-selectin, and anti-α-tubulin, and incubated overnight at 4 °C. Finally, horseradish peroxidase secondary antibody conjugates were reacted with the membranes for 1 h and detected by enhanced chemiluminescence using a MultiGel-21-C2 image system (Topbio, Taiwan). The optical density was measured using ImageJ software (Bethesda, MD, USA).

### 4.2. Assay of Fucoidan Binding to the P-Selectin Protein and Cellular Uptake of Fucoidan

The commercial fucoidan was purchased from Marinova^®^ Pty Ltd. (Cambridge Hobart, TAS, Australia). The biological source of this fucoidan was Fucus vesiculosus. To investigate the effect of fucoidan on the selectivity of P-selectin, the reaction between the carboxyl group of fucoidan and the amine group of RD6G was used to synthesize fluorescent RD6G-conjugated fucoidan (RD6G-fucoidan). First, RD6G (0.5 mg/1.0 mL) dissolved in acetonitrile was dropped into an aqueous solution of fucoidan (25.0 mg/5.0 mL) in deionized water until thoroughly mixed. Subsequently, 1-(3-Dimethylaminopropyl)-3-ethylcarbodiimide hydrochloride (1.0 mg) was added to the solution with continuous stirring overnight at 4 °C. To remove unbound reagents and fluorescent dyes, RD6G-fucoidan was dialyzed against deionized water until fluorescence was undetectable in the supernatant, and powdered RD6G-fucoidan was obtained after freeze-drying.

To verify the ability of fucoidan to bind the P-selectin protein, recombinant P-selectin (0.5 µg/50 µL) was added to a hydrophobic enzyme-linked immunosorbent 96-well plate and incubated overnight at 4 °C [45]. Wells were washed with PBS and incubated with bovine serum albumin (0.1 mL/well) for 1 h. The wells were then carefully washed with PBS again and co-incubated with fluorescent RD6G-fucoidan of varying concentrations (0.05, 0.10, 0.20, 0.40, and 0.80 mg/mL) for 30 min. Finally, each well was washed with PBS and the fluorescence intensity (excitation 525 nm/emission 555 nm) was measured using a Thermo Scientific™ Varioskan™ LUX multimode microplate reader. Cellular uptake by DU/DX50 cells treated with fluorescent dye-conjugated RD6G-fucoidan was assessed by flow cytometry. Then, RD6G-fucoidan solutions at varying concentrations (0.05, 0.10, and 0.20 mg/mL) were incubated with DU/DX50 cells for an additional 24 h, at 37 °C. Untreated cells were used as controls. The cells were washed with Dulbecco’s phosphate-buffered saline (DPBS), and 0.5 mL DPBS was also used to suspend and collect the cells. The intracellular RD6G-fucoidan content was evaluated and investigated using a Becton Dickinson FACSCalibur™ flow cytometer and CellQuest™ Pro software Version 5.1 (Verity Software House, Inc., Topsham, ME, USA).

### 4.3. Determining the Binding Ability to P-Selectin and the Effect on the Expression of Related Proteins in Fucoidan-Treated PCa Cells

To observe the distribution of fucoidan and its ability to bind the P-selectin protein in cells, DU/DX50 cells (2 × 10^5^/mL) were seeded on glass coverslips and allowed to attach for 24 h. The growing cells were treated with different concentrations (0.00, 0.10 and 0.20 mg/mL) of fluorescent RD6G-fucoidan solution for 24 h and washed three times with DPBS. They were then fixed in 1.0% paraformaldehyde, permeabilized in 0.1% Triton X-100 for 15 min, and blocked with 1.0% BSA in PBS for 1 h. Subsequently, the specimen was incubated with diluted primary mouse anti-P-selectin antibodies, at 4 °C, overnight. After washing with PBS, the samples were further preserved with secondary anti-mouse Cy5^®^ antibody for 1 h in the dark, and the nuclei were stained with 4’,6-diamidino-2-phenylindole (DAPI) and mounted on glass slides. These samples were examined by CLSM followed by MetaMorph software analysis to quantify the fluorescence images [46]. Moreover, DU/DX50 cells were incubated with the tested fucoidan samples, and the effects of multidrug-resistance-related proteins (ABCC1 and ABCB1) and P-selectin protein expression were assessed by Western blotting, followed the by measurement of optical chemiluminescence density using ImageJ software.

### 4.4. Determination of the Effect of Fucoidan on the Migration and Invasion of Docetaxel-Resistant PCa Cells

To analyze the effects of fucoidan on cell migration and invasion, docetaxel-resistant DU/DX50 cells were cultured at a density of 1.0 × 10^6^/5 cm for 24 h and incubated with different concentrations (0.000, 0.025, 0.050, 0.100, and 0.200 mg/mL) of fucoidan, at 37 °C, for 6 h. The treated cells were washed with pre-warmed DPBS and harvested with trypsin-EDTA solution for future experiments. The collected cell pellet (1.0 × 10^4^/chamber) was then resuspended in serum-free medium (0.5 mL) and added to the upper compartment of the Transwell insert for migration. A Matrigel-coated Transwell insert was added to the invasion chamber, then 1.0 mL of medium with 10% FBS was added to the lower compartment. Following an additional 16 h incubation at 37 °C and 5% CO_2_, the migrated cells on the lower surface of the filter were fixed by 3.75% paraformaldehyde and stained with 0.2% crystal violet, at room temperature. Five fields of view were randomly selected from each well and photographed using a light microscope (100× magnification), and the number of cells was counted.

### 4.5. Analysis of the Effect of Fucoidan on the Expression of Metastasis-Related Proteins

To analyze the effect of fucoidan on the expression of metastasis-related proteins in docetaxel-resistant PCa cells, the cells were treated with fucoidan for 24 h. Whole cell lysates of DU/DX50 cells were lysed and harvested with microwestern array (MWA) lysis buffer (Tris-acetate (240 mM), EDTA (5 mM), SDS (1%), glycerol (0.5%), fresh protease inhibitor cocktail, phosphatase inhibitor cocktail, and sodium orthovanadate (1 mM)). MWA was performed to detect different protein-expressing antibodies, as previously described [47]. The α-Tubulin and β-actin were used as loading controls. Scanned images were acquired using the Odyssey Infrared Imaging System. The band intensities of different expressed proteins were quantified using the Image Studio software (version 5.2; LI-COR Biosciences, Lincoln, NE, USA).

To quantify the effect of fucoidan on the expression of metastasis-related proteins by traditional Western blotting, the cells were lysed using SDS lysis buffer containing dithiothreitol, a cocktail of phosphatase inhibitors, and protease inhibitors. Antibodies against IL-1R, IKKα, and NF-κB p50 were obtained from Santa Cruz Biotechnology (Dallas, TX, USA). Antibodies against Cox2 were purchased from Cell Signaling Technology (Danvers, MA, USA). GAPDH and ꞵactin antibodies were purchased from Novus Biologicals (Littleton, CO, USA) and used as internal controls for equal loading. Signals in immunoreactive blots were detected using enhanced chemiluminescent immunoblotting substrates.

### 4.6. Evaluation of the Anticancer Effect of Docetaxel-Resistant PCa Cells and the Determination of Combination Index

DU/DX50 cells were seeded in 96-well plates at a density of 1 × 10^4^ cells/well. Following overnight incubation, the cell medium contained various concentrations of fucoidan (0.0–0.8 mg/mL), docetaxel (0.0–320.0 ng/mL), or fucoidan/docetaxel (various fucoidan concentrations ranging from 0.0–0.6 mg/L mixed with docetaxel concentrations of 160.0 or 320.0 ng/mL) solutions. The additive effect of the fucoidan/docetaxel combination on the viability of drug-resistant cancer cells was determined according to the Chou–Talalay method. The combination index (CI) was calculated using the following equation: CI = D_F_/D_xF_ + D_D_/D_xD_. D_F_ and D_D_ are the concentrations of fucoidan and docetaxel, respectively, used to achieve x% drug efficacy in the combination therapy. D_xF_ and D_xD_ are the concentrations of fucoidan and docetaxel, respectively, used alone to achieve an x% drug effect. CI < 1.0, CI = 1.0, and CI > 1.0 indicate the synergistic, additive, and antagonistic effects of the combined agents, respectively [48,49]. The correlation between the drug concentration and inhibitory concentration (IC), which inhibits cell viability reduction, was used to create isobolograms and combination index–fraction-affected (CI-Fa) plots.

## 5. Conclusions

In this study, we elucidated the biological activities and functions of fucoidan, which has important implications in the metastasis of docetaxel-resistant cancer cells. Fucoidan attenuated the motility of docetaxel-resistant DU/DX50 cells by binding to P-selectin and decreasing its expression, resulting in the downregulation of IL-1R, inactivation of NF-κB, and reduction in Cox2 expression. We also observed the synergistic effect of fucoidan and docetaxel on the viability of docetaxel-resistant PCa cells.

## Data Availability

Not applicable.
